# History, concepts and aims of internationally active societies in psychosomatic and behavioural medicine

**DOI:** 10.1186/s13030-016-0085-1

**Published:** 2016-12-12

**Authors:** Hans-Christian Deter

**Affiliations:** Charite Universitatsmedizin Berlin, Berlin, Germany

Many scientists and practitioners from different countries and organizations are working in the field of Psychosomatic and Behavioural Medicine. The clinicians are looking for a better health care and are trying to increase their quality in an individual patient. The scientists are trying to understand the interaction in a patient between mind, body and social environment.

Different countries and cultures experienced different developments. This has an impact on the present situation in theory and practice of their medicine. To compare these activities in an overview over different societies from Asia, America and Europe could be provide an example for many others, active in this medical field. Especially their aims and future perspectives can be focused to understand the complexity of ideas and thoughts in these groups of physicians, psychologists and sociologists - and their different points of view. Following these perspectives, the diagnostic evaluation of diseases with their bio-psycho-social background can be demonstrated. The understanding of their psycho social mechanism leads to different treatment options. Not surprisingly these depend on the knowledge and their cultural meaning of triggering and influencing factors of such diseases.

In the following series we offer an overview, what are experiences and perspectives in selected national (countries with a great membership and long history) and internationally active societies of psychosomatic and behavioural medicine. We describe their ideas, aims and activities. The reader will get an understanding and an overview about fields, tasks and hopefully he/she will be stimulated for activities and cooperation, which are necessary in the future of Psychosomatic and Behavioural Medicine. Well known authors and members of the respective societies describe crucial events in their history, concepts and aims, which were now summarized in the following papers:

1. J. Streltzer presented his personal experiences he made during his time as member, commission chair and president of the International College of Psychosomatic Medicine.
2. M. Murakami and Y. Nakai, presidents of the Japanese Society of Psychosomatic and Internal Psychosomatic Medicine, summarized the basics, development and aims of this Asian psychosomatic society.
3. H.C. Deter, K. Orth-Gomér, B. Wasilewski and R. Verissimo, former presidents of the German College of Psychosomatic Medicine, the International Society of Behavioural Medicine and the Polish Psychosomatic Society described an initiative to develop activities in a European Network on Psychosomatic Medicine
4. S. Zipfel, H.C. Deter, J. Kruse, actual and former presidents of the German College and the German Society of Psychosomatic Medicine describe the German history, actual situation of Psychosomatic Medicine and the chances of psychotherapeutic specialization in German physicians.
5. K. Orth-Gomér and N. Schneiderman, founding members and former presidents of the International Society of Behavioural Medicine, describe the environment, psychobiological mechanism, psychological processes and public health as main topics of behavioural research activities.
6. Ch. Herrmann-Lingen and D. Drossman, actual and former presidents of the American Psychosomatic Society present their history, new ideas and results of the APS strategic planning group.

Historical aspects, concepts and aims of psychosomatic and behavioural societies should be outlined and the barriers for implementation of old and new psychosomatic ideas. Influencing factors to increase the quality of a national health care system should be discussed and in which way psychosomatic societies in the present shape are willing to do it or - if so - can be successful.

## Historical aspects

Psychosomatic Medicine is an old concept and in its organizational dimension, a relatively new discipline. Against other disciplines its content is broad and wide and reaches from one disease to the whole field of medicine. Different traditions in psychosomatic medicine in Europe, Asia and America have lead to different cultures in medicine, including tendencies to define psychosomatic medicine as discipline with C/L psychiatry or psychotherapeutic medicine as focus.

### The origin of the name

The cultural history and cultural background as well as the personal history are important for the development of psychosomatic medicine in a country [1]. In Europe this name was created 1818 by J. Heinroth in Leipzig, the French philosopher R. Decartes had described 150 years earlier the differences between body and soul and English physicians had described in this time diseases in a very psychosomatic way. Since centuries the Asian culture focused on different biological, psychological, environmental and ethical aspects of a life in harmony and for an attempt to harmonise human beings. The American way of life in psychosomatic medicine developed from psychoanalytical roots to targeted, effective and cognitive structured research activities and became a pacemaker for research activities in behavioural and psychosomatic medicine. This research paradigm seems to push back other cultural and personal experiences in the field. So it is interesting to compare these traditions and scopes of psychosomatic medicine in different countries: Paper 5 and 6 of this series presents this American way of structural thinking, comprehensive research hypotheses and managing successful good research projects. Paper 2 presents the Japanese ideas of psychosomatic harmonization in an individual with the surrounding of family, society and environment under an ethical aspect. Impressive is the effort, which was made to organize successfully psychosomatic structures in Japan. Paper 4 described the historical development of psychosomatic medicine in Germany and the concept of different psychotherapeutic interventions on all levels of health care. All these papers are written from members within a society which has members, website and a members only section (see below). Paper 3 discussed the advantages of a free cooperation of all interested scientists or clinicians, what was intended within the European Network of Psychosomatic Medicine.

### Concepts and recent development

The concepts of psychosomatic and behavioural medicine have similarities and differences, if you compare the definitions of Kimball/Engel and Schneiderman/Orth-Gomér: Psychosomatic medicine is in view of Kimball [2] “All illnesses have psychosocial aspects that influence their cause, precipitation, manifestation, course and outcome.” “The approach to the individual suffering from a specific illness is specific depending on the idiosyncrasy of the patient’s life situation, which includes, in addition to attending to the disease process, attending to the psychological and social correlates.” In G. Engel’s (1979) definition correspond psychosomatic medicine with bio-psycho-social medicine and means on one hand a holistic dimension of medicine. On the other hand it explains in a scientific way differentiated bio psycho social mechanisms of etiology, course of somatic and somatoform diseases and applies possible intervention options [3].

“Behavioural medicine is (according to [5]) the interdisciplinary field concerned with the development and integration of biomedical, behavioural, psychosocial, and socio cultural science knowledge and techniques relevant to the understanding of health and illness, and the application of this knowledge to disease prevention, diagnosis, treatment, rehabilitation and health promotion.” It seems, that psychosomatic medicine is closely related to the clinical reality of in- and outpatient care and to the individual patient. Behavioural medicine has a broader scope especially for public health questions, human behaviour and shows less interest in the individual patient, case reports or integrated clinical care, but more on high quality treatment studies with acceptable sample sizes. In this series 5 paper were written from a psychosomatic view (paper 1–4, 6) and one from the behavioural view (paper 5). Consultation liaison services [4] and practice questions of clinical care were interesting for 4 societies, three for psychiatric C/L (paper 1–3) and three for psychosomatic C/L services (paper 2–4).

If we focus the scientific, sociological and medical frame work of psychosomatic medicine in the last decades, several important changes in the society and their subsystems emerged. So traditional concepts have changed (new definition of APS; 6. Herrmann-Lingen&Drossman in this series) and activities have to be reviewed in different fields. This leads to new tasks, which are seen in the presented papers similar (in brackets), or different.

*Aims of psychosomatic and behavioural medicine:*

1. *Psychosomatic and Behavioural Medicine have to grow and spread out (paper 1,3,5):* New societies were founded in different countries and cultures. Members of these societies are not only physicians, now societies integrate different professions, health care providers, in an interdisciplinary way. They have different needs and aims, and will benefit in their actions and organisational activities from others (1. J. Streltzer in this series). This development should go on.
2. *Evidence for a theoretical basis of Psychosomatic Medicine should increase (all papers)*: In the last 80 years, there was a huge increase in medical and psychosomatic knowledge, the last 30 years were overwhelming - also through the research activity of the societies presented in this series - according to scientific advancement in many diseases (6. Hermann-Lingen & Drossman in this series). That had led to a high specialization in many fields and research groups, e.g. the neuroscience - brain, mind and body research has brought new important perspectives. In this series the main focus on clinical research and therapeutic outcome studies was seen in paper 5 and 6, but also in paper 2 and 4, research in Public Health only in paper 5 and 6 and less experimental basic research in paper 2 and 4.
3. *Better understanding of research organization and funding* (*paper 3–6*) *–in a national and international perspective*: In the scope of funding organizations psychosomatic and behavioural medicine is a small discipline, when it applies for research grants in medicine on a national or international level. The competition with disciplines of the basic sciences like genetics, brain research or immunology is high. It is necessary to cooperate with psychiatry, psychology and the somatic disciplines in different fields and diseases. An attempt to define a special claim and a speciality may have limited success. In a global perspective there is a decrease of funding in human and cultural sciences compared to basic natural sciences, which seem more cost effective and promise more economic benefit.
4. *Applying more psychosomatics in clinical practice* (*paper 2–4*): GP or the internist/somatic specialist treat patients with somatic diseases and psychosocial factors, which influence symptoms or the course of disease e.g. coronary heart disease (CHD), diabetes, or asthma. Additionally they treat patients with somatoform disorders, anxiety, depression and somatic symptoms. They also care for somatic patients with psychiatric co-morbidity like cancer and depression – this field is organized very effectively by the Japanese Psychosomatic Society (2. Murakami & Nakai in this series). Which patients should be treated additionally by a physician or psychologist, psychotherapist, CL-psychiatrist, nurse, or health care worker trained in psychosomatic/psychotherapeutic skills for specific treatments? What could be the targets of these treatments: symptom minimizing, change of behaviour or personal psychological structure, change of conflicts in the family or at the working places? In standard care many psychotherapeutic treatment models were created by the German psychosomatic group (4. Zipfel et al. in this series).
  In this time we are confronted in many countries also with other options: What are the advantages of a psycho pharmaceutical intervention before, beside, after or instead of a psychotherapeutic treatment? What about traditional medicine (Chinese, Japanese, European)? Should it be applied in special cases? The personality of physician, psychologist, or nurse and his/her influence on the patient has proven to be extremely important for the therapeutic process. These non specific influencing factors have to be detected [6] beside other factors, being effective in a specific psychotherapeutic technique.
5. *Communication in the medical field with other specialities and societies is necessary* (*all papers*): Is there a need to communicate to focus on the own interests, methods and fields? Who is interested to learn from each other? Two examples should be given: a. Specialist centre for different diseases like diabetes or asthma are interested to get knowledge and expertise from psychosomatic or behavioural experts in the field to use it for their patients. Such experts are welcome and helpful for clinical, but also for research reasons. b. The development of national or international guidelines for diagnostics and therapy of different diseases are a good basis to communicate between different specialities and research fields. The 3rd to 6th task forces of European Guidelines in cardiovascular disease prevention are a good example for effective co-working of eight medical societies, including the International Society of Behavioural Medicine ([8]; 5. Schneiderman & Orth-Gomér in this series).
6. *Increase of psychosomatic health care and more activities in health care systems* (*paper 3–5*): What is the adequate, acceptable and cost effective psychosomatic care for patient groups in an epidemiological perspective? What are the right indicators: hard endpoints like mean age at death, mean disability adjusted life years (DALY) or quality of life of a population? We are only on the way to answer these questions for some diseases, but not for all. Is there a standard care according to psychosomatic issues necessary or does it depends on from physicians experience with the special patient group, on the outcome of evaluation processes in a special health care system, cultural traditions, or on national wealth [9]?
7. *Care for health care professionals* (*no paper*): Earning sufficient money for psychosomatic professionals to support their spouses and children, is another essential for increasing psychosomatic activities in the national health care system.
8. *Improving psychosomatic training* (*paper3,4*): Teaching communication skills and physician-patient relationship interaction patterns on a cognitive, behavioural, emotional and physiological level (European Association in Communication and Healthcare, Bensing et al.2011). The art and science of communication can take place on different levels: student, GP, C/L-Psychiatry, Internal medicine, other specialities, or psychology [7].

### Requirements for reaching the psychosomatic and behavioural aims

*- knowing the barriers - implementation of aims is in competition*:

- *between all medical disciplines in the medical faculty* in a university according to reputation in research activities, e.g. scientific output, impact factors, research positions in the university and in getting research money/grants. The basic sciences are struggling with the clinical sciences and with public health activities. In the last years research foundation like the German research foundation spent most of their funds for genetic, molecular and high technological research projects; and pharmaceutical companies spent their money for new pharmaceuticals. So Behavioural and Psychosomatic Medicine is compared to other disciplines in an inferior position outside of the US (funding of National Institute of Health (NIH) in the Behavioural Medicine branch for cardiovascular disease).
- *according to the influence in the medical societies/administrations/insurances* in each country, which is responsible for the health care expenses.
- *between different medical specialities* like internal medicine, gynaecology, psychiatry etc. Here it is important, which health care field specialities try to claim for themselves. E.g. there is a long standing discussion, if functional GI-disorders like the irritable bowel syndrome belong into internal medicine or - as somatoform disorder - into the psychiatric field [10]. Or if psychological symptoms, e.g. in patients with asthma, are a challenge for the internist, the psychosomatic internal specialist or – as “depression/anxiety of the medical ill” for the C/L psychiatrist. This depends on severity of symptoms and the real health care situation in a country (3. Deter et al. in this series). Who has the arguments, competence and the power in a given society to occupy this field?
- *between different professions*: *Physicians, psychologists and nurses* as well other health care professionals. Why do we have different associations or societies dealing with psychosomatic medicine? The main reason is that three different professional groups are doing research in the field of psychosomatic medicine: psychiatrist, psychologists and physicians in internal medicine or other specialties with or without additional training in psychotherapy. Partly, those researchers have different interests and agendas. It is by no means obvious that an overriding society can include all those aspects simultaneously [11].

*- knowing the cues of how to increase the quality of a national health care system and in which way psychosomatic and behavioural medicine can reach their aims.*

If psychosomatic and behavioural medicine will be successful in these different, national and international fields, depends on several factors:

- Evidence of psychosomatic research according to diagnoses and intervention in different diseases. This leads to the implementation in national and international guidelines for each different disease.
- Here it is useful to know, that clinical experience is mostly found in physicians (internists, psychiatrists), nurses and physiotherapists, research activities in physicians (neurologist, psychiatrist, internist), but often more in psychologist (psycho physiology, psycho neuro immunology) and public health questions more often found in epidemiologists, sociologists and health psychologists.
- A continuous collaboration in a special clinical field over ten or more years (like cardiologists and specialists in behavioural medicine in preparing the European Guidelines of Cardiovascular disease prevention [8, 12]
- A longstanding personal communication between representatives of psychosomatic/behavioural and representatives of a clinical specialty or working group in a special clinical field with participation in clinical scientific meetings and special working groups and common publication of own studies
- Cooperation with patient initiatives (self help organizations) and the public (newspapers, television, internet communities)
- A cooperation with health care politicians, which was demonstrated in the speech of the Australian ministry of health during the International Conference on Behavioural Medicine in Brisbane [9], as well as with national and communal health care organizations. Highlights in this sense were the speech of the Queen of the Netherlands at the World Conference for Psychosomatic Medicine in Amsterdam1973 or the invitation of the Japanese Emperor through Japanese psychosomatic physicians for the World Conference of Psychosomatic Medicine in Kobe (2. Murakami & Nakai in this series).
- Health care and political aims can only be fulfilled, if there is a group of physicians, psychologists and others, interested in research and clinical practice and they fertilize with their ideas, activities and common actions in the field of psychosomatic/behavioural medicine.

*- Do we have sufficient psychosomatic organizations for the present task? – In which way can they help to reach these aims in a given society?*

Earning of money, reputation and power within a society, national and international, depends on the influence of the member of this group within the medical organization, medical faculty and the health care system.

### Members

Interested, active and open minded members are the basis of a society. The interest for a society depends on the visibility in the scientific community, on the website, at newspaper or television, and from society’s history. It is important, where the members come from, which profession, which experiences they have and what are their individual interests and aims they want to reach with their membership: new experiences, research questions, information about new care options; discussing with people brings new information and contacts. Not to underestimate is the professional interest to reach better professional conditions/positions/influence in the own faculty (career development). Another motive may be an idealistic one: to make medicine better in a psychosomatic/behavioural way.

### Group identity

Working together for the same targets as basis for a working group or psychosomatic national society. It needs good relationships and the possibility to co-work with each other.

### Function of annual or biannual scientific meetings

The conference has an important function of a society, it is a “market place”, where society members and other scientists present their research, thoughts and ideas in the psychosomatic/behavioural field. An interactive discussion of these proposals and results stimulates new ideas and research activities. The number of participants, presentations, posters is an indicator for the success of the society. There is a discussion, if a famous and most experienced scientist/clinician in the psychosomatic field (ECPR, ICPM) should be responsible for the conference or a program committee elected by the board of the society (ISBM, APS) or a scientist/clinician living in an exciting venue country/city. The innovative scientific and clinical output of a conference influence the visibility of a society, their thinking and their future activities.

### Communication within the society

There are several models for a discussion of topics within a society beside the scientific annual or bi-annual conferences (which could be supplemented by additional scientific meetings focused on a special topic) and the business meeting of the society. The information (minutes) about all meetings of board, committees, general assembly and other activities of the society including conference program and abstract books are seen at the members only section of the website. Additionally a newsletter spread once or twice a year will support the communication between members. Committees, working and special interest groups meet each other on the conferences and in between. They could work with or without a program and aim to cooperate or coordinate clinical or research questions. They are an important indicator for the activity of a society beside conferences and journals.

### Function of the scientific journal, its editor and its editorial board

They decide about: what is research quality, what should/can be published. They organize the direction of interest within the psychosomatic field and get the response by quality of submitted research and Impact Factor. So this group is very influential for the success of a society.

### Power and democratic mechanisms within a psychosomatic society

The president, vice president, board members, chairs of committees and working groups are in intensive communication and they can influence within their limited active period the actual situation and the way a society takes. In some societies there are strategic and planning committees (ISBM, APS), which foster ideas, aims and milestones for society development and present them to the board. If these will be realized, a majority of the board or some of its influential members decide; this depends on many factors within and outside of a society.

### Cooperation with other societies in the medical and psychosomatic field

It is interesting, that each society has a tendency to look first on own issues. Often there is no great interest to cooperate with other societies. There are needs to concentrate on own members, tasks, by laws and activities of the society. There is less space for national networking or for an integration and work with other societies in clinical medicine.

### Future perspectives

Creativity is needed, development of new research questions and arguments for doubts. It has to be possible to modify the own perspective (in a sense of a dialectic process: thesis-antithesis-synthesis). Communication with others working in the field is helpful - with friends and cooperation partners, but also with “non communication partners or competitors”. A common language is sometimes difficult but useful.

## Conclusion

So it seems interesting to summarize some of the ideas, activities and aims, which different national and international societies in psychosomatic and behavioural medicine have developed. The reader can compare different aspects and can get an own opinion, about what is going on in the psychosomatic/behavioural scene, what aims are helpful and what activities should been made. All of these societies had different histories, aims and concepts, they and many others should talk together and figure out how to have more of an influence on national health care, common research projects and scientific funding agencies. There was no interest in exploring whether the different international societies could join forces, becoming an umbrella organization and speak with a common voice. The six societies presented here are only a selection of many others, e.g. Academy of Psychosomatic Medicine, Society of Health Psychology, Society of Behavioural Medicine, International Society of Psychophysiology, Society of Psycho-neuro-immunology, Society for Neuroscience, national PSM/BM societies, different psychosomatic/psychiatric branches of specialist societies, etc. They all may have different ideas and experiences, but all working on psycho-somatic aspects in the medical field. In this situation, we want to summarize for patients, physicians, psychologists and other health care professionals the actual situation in the field, which could be supplemented by others in one of the next series of BioPsychoSocio Medicine.
